# Adenosine and Inflammation: Here, There and Everywhere

**DOI:** 10.3390/ijms22147685

**Published:** 2021-07-19

**Authors:** Silvia Pasquini, Chiara Contri, Pier Andrea Borea, Fabrizio Vincenzi, Katia Varani

**Affiliations:** 1 Department of Translational Medicine, University of Ferrara, 44121 Ferrara, Italy; psqslv@unife.it (S.P.); chiara.contri@unife.it (C.C.); vrk@unife.it (K.V.); 2 University of Ferrara, 44121 Ferrara, Italy; bpa@unife.it

**Keywords:** adenosine, inflammation, adenosine receptors, immune system, chronic inflammatory diseases

## Abstract

Adenosine is a ubiquitous endogenous modulator with the main function of maintaining cellular and tissue homeostasis in pathological and stress conditions. It exerts its effect through the interaction with four G protein-coupled receptor (GPCR) subtypes referred as A_1_, A_2A_, A_2B_, and A_3_ adenosine receptors (ARs), each of which has a unique pharmacological profile and tissue distribution. Adenosine is a potent modulator of inflammation, and for this reason the adenosinergic system represents an excellent pharmacological target for the myriad of diseases in which inflammation represents a cause, a pathogenetic mechanism, a consequence, a manifestation, or a protective factor. The omnipresence of ARs in every cell of the immune system as well as in almost all cells in the body represents both an opportunity and an obstacle to the clinical use of AR ligands. This review offers an overview of the cardinal role of adenosine in the modulation of inflammation, showing how the stimulation or blocking of its receptors or agents capable of regulating its extracellular concentration can represent promising therapeutic strategies for the treatment of chronic inflammatory pathologies, neurodegenerative diseases, and cancer.

## 1. Introduction

Inflammation arises from a complex interplay between immune cells and many soluble mediators with the aim to protect the organism from harmful stimuli such as microorganism infections, damaged cells, or irritants as well as being a strong component of many pathological conditions like rheumatic diseases, neurological disorders, and cancer [1]. Adenosine, a pervasive autacoid, is considered a key mediator of the immune response. In physiological and unstressed conditions, the extracellular concentrations of adenosine are maintained at a low level as a result of the rapid metabolism and uptake [2]. Nevertheless, its levels rise considerably during conditions involving increased metabolic demand, hypoxia, tissue injury, and inflammation regulating the immune response.

**Figure 1:**
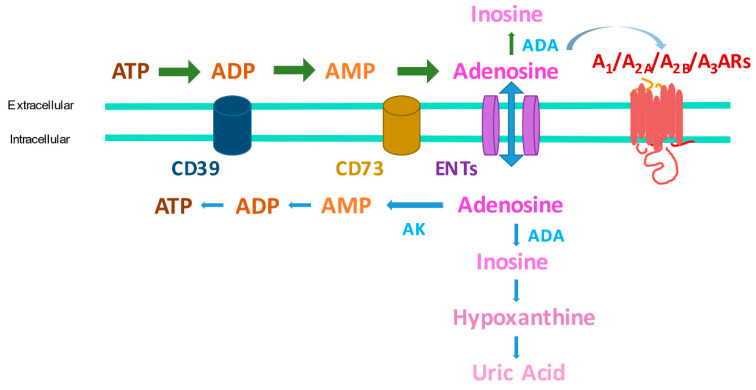
Adenosine metabolism and transport in the extracellular and intracellular milieu. Abbreviations: ATP: adenosine triphosphate; ADP: adenosine diphosphate; AMP: adenosine monophosphate; ADA: adenosine deaminase; AK: adenosine kinase; ENTs: equilibrative nucleoside transporters; ARs: adenosine receptors.

Adenosine exerts its functions through the interaction with four adenosine receptors (ARs), all of them are transmembrane G protein-coupled receptors (GPCRs) named A_1_, A_2A_, A_2B_, and A_3_ARs. Interestingly, the A_1_ and A_3_ subtypes are coupled to Gi proteins and have an inhibitory effect on adenylyl cyclase (AC) activity; while A_2A_ and A_2B_ARs stimulate it, through the coupling to Gs proteins, with a consequent modulation of cyclic AMP (cAMP) levels [3]. Adenosine production occurs through different mechanisms, the principal is by the dephosphorylation of the adenine nucleotides (ATP, ADP, and AMP) to adenosine. Especially ATP serves as the reservoir for the production of adenosine because it is the most abundant molecule in the cell [4]. Under resting conditions, some ATP is dephosphorylated to adenosine, but stressful stimuli can increase rates of intracellular conversion of ATP to adenosine. More commonly, these stimuli trigger the release of adenine nucleotides into the extracellular space where they are dephosphorylated to adenosine mainly by the combined action of two hydrolyzing enzymes termed ectonucleoside triphosphate diphosphohydrolase (CD39) and ecto-5′-nucleotidase (CD73) [3]. Once formed or released into the extracellular space, adenosine can be deaminated to inosine through adenosine deaminase (ADA) or taken up directly by cells by specific nucleoside transporters (ENT1 and ENT2) and re-phosphorylated to ATP [5,6] (Figure 1). 

Although there are instances in which adenosine exerts detrimental effects in various pathological conditions, it is generally considered a protective and homeostatic mediator against tissue damages and stress conditions [4,5]. In particular, adenosine presents immune-regulatory effects, mostly anti-inflammatory, which strengthen its general tissue-protective functions. In some cases, however, the effect of adenosine on the immune system is deleterious, as prolonged adenosine signaling may impede antitumor and antibacterial immunity, thereby promoting the development and progression of cancer and sepsis, respectively [1,7].

The focal point of this review is to describe the current knowledge about the role of ARs in inflammation, starting from the regulation of immune cells by ARs and then discussing their role in different pathological conditions where inflammation is predominant such as rheumatic diseases, lung and intestinal disorders, neuroinflammation, and cancer.

## 2. Regulation of Immune Cells

**Figure 2:**
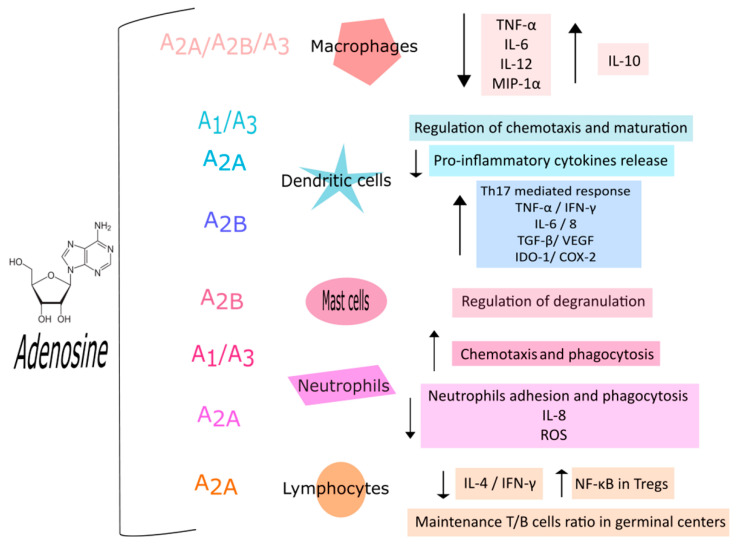
Schematic representation of main actions mediated by adenosine receptors (ARs) in human immune cells. Abbreviations: TNF-α: tumor necrosis factor α; IL: interleukin; MIP: macrophage inflammatory protein; IFN-γ: interferon γ; TGF-β: tissue growth factor β; VEGF: vascular endothelial growth factor; IDO-1: indoleamine 2:3 dioxygenase; COX-2: cyclooxygenase 2; ROS: reactive oxygen species; NF-κB: nuclear factor κ-light-chain-enhancer of activated B cells; ↑: increase; ↓: decrease.

Adenosine is considered a crucial mediator of the immune response. ARs are expressed in all kinds of immune cells where they participate in the regulation of immune and inflammatory responses, usually with anti-inflammatory effects supporting the protective role of adenosine (Figure 2) [1]. ARs are present on both monocytes and macrophages, with different expression levels depending on maturation advancement from monocytes to macrophages. In particular, in quiescent monocytes, a modest expression of A_1_, A_2A_, and A_3_ARs has been reported which rises over differentiation into macrophages [8]. Many studies also investigated the role of proinflammatory cytokines on AR expression reporting that interleukin (IL)-1 and tumor necrosis factor α (TNF-α) enhance both A_2A_AR expression and functionality, averting receptor desensitization, in human monocytes [9,10]. The anti-inflammatory effect of ARs in macrophages is supported both by A_2A,_ A_2B_, and A_3_AR activation and by extracellular adenosine. The Ars’ activation blocks the release of proinflammatory mediators like TNF-α, IL-6, IL-12, nitric oxide, and macrophage inflammatory protein (MIP)-1α [11,12,13] while adenosine, acting on A_2A_ and A_2B_Ars, endorses the release of IL-10, an anti-inflammatory cytokine [14,15,16]; while the A_3_AR stimulation promotes macrophages chemotaxis towards apoptotic cells [17]. Adenosine is also involved in the regulation of dendritic cells, the antigen-presenting cells able to trigger adaptive immune response [1,18]. A_1_ and A_3_ARs are mainly present in immature dendritic cells where they regulate chemotaxis through intracellular calcium rise. Differently, in mature dendritic cells mostly A_2A_ARs are expressed leading to a decrease in proinflammatory cytokines [19]. On the contrary, a proinflammatory effect of A_2B_ARs in dendritic cells has been reported. In fact, they have the capability to shift bone marrow cell differentiation to a specific subpopulation of dendritic cells which activate Th17 cell response [20]. Th17 cells play a role in host defense against extracellular pathogens, particularly at the mucosal and epithelial barriers, but aberrant activation has been linked to the pathogenesis of various autoimmune diseases [21]. Moreover, A_2B_ARs in association with ADA form a complex that interacts with CD26 on T cell surface leading to TNF-α and interferon γ (IFN-γ) release [22]. Other evidence, supporting the proinflammatory role of this receptor subtype, reports that the activation of A_2B_ARs moves dendritic cell differentiation to a proangiogenic and proinflammatory phenotype. In particular, in hypoxic conditions A_2B_ARs stimulation provokes the release of proangiogenic mediators such as IL-6, IL-8, IL-10, transforming growth factor β (TGF-β), vascular endothelial growth factor (VEGF), indoleamine 2,3 dioxygenase (IDO-1), and cyclooxygenase 2 (COX-2) [23].

Even though the role of ARs in monocyte, macrophages, and dendritic cells has been extensively studied, their role in mast cells remains poorly understood. Mast cells are an important component of the immune system and are present throughout the body playing crucial roles in the maintenance of many physiological functions, such as vasodilation, angiogenesis, bacterial, and parasite elimination, as well as in the pathophysiology of diseases. Moreover, mast cells regulate the functions of several cell types, including dendritic cells, macrophages, T and B cells, fibroblasts, eosinophils, endothelial cells, and epithelial cells [24,25]. Studies have reported that ARs are expressed in human skin mast cells; conversely, studies carried out in the human mast cell line LAD2 and HMC-1 cells demonstrated that both cell lines express A_2A_, A_2B_, and A_3_, but not A_1_ARs [26,27]. In murine mast cells, A_2B_ and A_3_AR activation prompts degranulation leading to histamine, serotonin, chemokine, and protease release [26]. Other studies in human mast cells confirmed that A_2B_AR activation is principally implicated in mast cell degranulation, while A_3_AR stimulation seems to mediate anti-inflammatory effects [28]. 

Adenosine also regulates the activation of neutrophils, which are pivotal modulators of both inflammation and immune responses. Neutrophils are the most copious leukocytes of the immune system with a great phenotypic heterogeneity and functional versatility [29]. Neutrophils also represent a large source of adenosine in particular in inflammatory conditions when they release ATP that is rapidly converted into adenosine through CD39 and CD73 expressed on neutrophils surface [30,31]. Notably, inflammation reduces adenosine metabolism through ADA deactivation, and equilibrative transporters decrease [30]. Studies have demonstrated that all four AR subtypes are expressed in neutrophils [25]. In particular, A_1_ARs promote neutrophil chemotaxis, whereas A_2A_ and A_2B_ARs inhibit neutrophil activation [32]. In fact, treatment with the A_2A_AR agonist, ATL313, inhibits critical steps in integrin-dependent neutrophil adhesion in vitro and in vivo [33]. A subsequent study, investigating the signaling under the inhibitory function of A_2A_ARs stimulation in neutrophils, reported that following treatment with the A_2A_AR agonist CGS 21680 the phosphorylation of p38 MAPK, Erk-1/2, PI3K/Akt, HCK, and SYK protein kinases was decreased [34]. Furthermore, A_2A_ARs dampen IL-8 release, impairing neutrophil degranulation [35]. The activation of A_1_ and A_2A_ARs is also involved in neutrophils phagocytosis with an opposite role: A_1_ARs increase phagocytosis while A_2A_ARs reduce it. Even the A_3_AR agonist 2-Cl-IB-MECA promoted the formation and rapid extension of projections that improve bacterial phagocytosis and chemotaxis [36]. Moreover, adenosine has a different effect on reactive oxygen species (ROS) generation based on the receptor subtype activated [31]. In particular, the stimulation of A_1_ARs induces ROS production from activated neutrophils, whereas the activation of A_2A_ARs down-regulates ROS generation [35]. A_2B_ or A_3_AR agonists suppressed stimulus-induced superoxide production in wild-type but not in A_2B_ or A_3_AR deficient neutrophils [37]. 

The cells responsible for cell-mediated immune response are T lymphocytes. These cells are triggered by antigen-presenting cells such as dendritic cells or macrophages evoking T cell differentiation, cytokine production, and cytotoxic activity [38]. ARs can forge many lymphocyte functions. A_2A_AR stimulation blocks IL-4 and IFN-γ production in both naive CD4+ T cells and Th1 and Th2 cells [1]. Recently, a crucial role of A_2A_ARs in maintaining T follicular help cell/T follicular regulatory cell ratios including the overall ratio between T to B cells into the germinal centers has been reported [39]. Regulatory T cells (Treg) are a subpopulation of T lymphocytes with the task to control and suppress autoreactive T cells preventing autoimmunity [40]. Tregs produce adenosine, which stimulates A_2A_ARs thus reducing proinflammatory cytokine release via nuclear factor-κB activation [41]. The adenosine generated by Tregs acting on A_2A_ARs activates Tregs cell expansion leading to additional immunosuppression with a self-reinforcing loop [42]. Recently, a critical role of the adenosine system in the modulation of B cell functions has been pointed out. ARs are expressed in both murine and human B cells, as well as the complex network of ectoenzymes (nucleotidases, deaminases, kinases) and nucleoside transporters [43]. Adenosine seems to be involved in regulating the development, implantation, and maintenance of the plasma cell population in bone marrow for the primary immune response but also in managing immunoglobulin class switching [1]. In particular, in inactivated B cells there is a higher extracellular concentration of adenosine, while once activated their ATP release increases. This mechanism seems to defend activated B cells from the adenosine-induced inhibitory effect and exerts a proinflammatory role [44]. Interestingly, as observed with Treg cells, adenosine can regulate the function of Breg cells, a subset of immunosuppressive cells that support immunological tolerance. In particular, Bregs were able to regulate both their own function and T cell activity via an adenosine signaling originating from the enzymatic degradation of ATP, released in the extracellular space from activated immune cells [45].

## 3. Rheumatic Diseases and Osteoarthritis

Rheumatic diseases are characterized by inflammation of the joints, ligaments, tendons, bones, or muscles and which in some cases can also involve other organs; several studies have shown that both the innate and adaptive immune systems can contribute to the inflammation seen in diseases such as rheumatoid arthritis (RA) and psoriatic arthritis [46]. Inflammation of the synovial membrane is characterized by the infiltration of leukocytes [47] leading to changes in endothelial permeability and increasing the adhesion of immune cells, releasing ATP [48]. In response to the increase in ATP concentrations, there is an increase in the activity of ectonucleotidases (enzymes that metabolize nucleotides into nucleosides) to control the inflammatory response and promote the formation of adenosine [49]. Adenosine production has emerged as an important cellular mechanism for regulating inflammation in chronic rheumatic diseases [50].

Methotrexate has been used in the treatment of RA for decades and is often the first-line medication for RA treatment. It is well known that methotrexate increases adenosine levels, and its induction of adenosine signaling is probably the most widely accepted explanation for the methotrexate mechanism in RA [46,51]. ARs are expressed in a large number of cells, including those involved in the pathology of RA such as lymphocytes, neutrophils, macrophages, and synovial cells, where they have predominantly anti-inflammatory effects [52]. Several studies demonstrated greater expression of A_2A_AR and A_3_AR receptors on lymphocytes and neutrophils isolated from RA patients compared to healthy subjects. No changes were observed in A_1_ or A_2B_AR [53,54,55]. The main anti-inflammatory effects of adenosine occur through the stimulation of A_2A_AR and A_3_AR, the expression of which is dynamically regulated by proinflammatory cytokines [56]. Up-regulation and stimulation of A_2A_AR and A_3_AR in peripheral leukocytes of RA patients inhibits the NF-κB pathway by decreasing IL-1β, IL-6, and TNF-α [54]. However, the expression of A_2A_AR and A_3_AR is normalized when anti-TNF agents are administered, demonstrating that their expression varies with inflammatory levels during RA [53]. Treatment with A_2A_AR agonists has been shown to increase serum levels of IL-10, an anti-inflammatory cytokine, and reduce the characteristic signs and symptoms of the disease in two different animal models of arthritis [57,58]. In the model of collagen-induced arthritis, stimulation of A_2A_ARs by a CD73-dependent prodrug markedly reduced joint inflammation [59]. In support of the importance of CD73 as a source of adenosine in arthritis, CD73-deficient mice were found to be significantly more susceptible to collagen-induced arthritis than wild-type mice [60]. 

CD4 germinal center (GC)-follicular helper T (Tfh) cells have an important role in the pathogenesis of autoimmune diseases [61,62]. In an interesting recent study using a mouse model of autoimmune arthritis, A_2A_AR stimulation diverts autoreactive CD4 T cell differentiation away from the GC-Tfh cell lineage, thus reducing the induction of autoreactive B cells that promote arthritis [63].

In different animal models of arthritis, the A_3_AR agonist CF101 exerted significant antirheumatic effects reducing the clinical and histological features of arthritis mainly by a decrease of TNF-α production [64]. Similar effects were obtained with the A_3_AR positive allosteric modulator LUF6000 through deregulation of NF-κB [65]. Another A_3_AR agonist, CF502, induced an inhibitory effect on the proliferation of fibroblast-like synoviocytes from RA patients and in adjuvant-induced arthritis rats and suppressed the clinical and pathological manifestations of arthritis in the rat model [66]. 

Despite promising results with CF101 in animal models, the results of early clinical trials have not demonstrated with certainty its ability to improve the course of RA in human patients [67]. Later, in two phase II clinical studies where CF101 was administered to RA patients as a stand-alone drug, a significant antirheumatic effect was observed. Furthermore, a direct significant correlation between receptor expression at baseline and patients’ response to the drug was found, suggesting that A_3_AR may be used as a predictive biomarker [68].

A_2B_AR’s role is not entirely clear and current research findings seem ambiguous. While some studies indicate that Th17 differentiation is stimulated by increased production of IL-6 in dendritic cells [69], others argue that A_2B_AR, when stimulated, promotes T cell differentiation into Treg [67]. The effects of A_2B_AR binding on osteoclast differentiation and bone resorption are also controversial. Researchers observed that the A_2B_AR agonist inhibits RANKL-induced osteoclast formation in mouse cells [70]. On the other hand, one study found that adenosine via A_2B_AR abolished the osteoclast inhibition induced by methotrexate [71]. A_1_AR stimulation, in turn, is essential for the bone remodeling process, through the differentiation of giant cells into osteoclasts during RA [72]. 

ADA levels increased in plasma and synovial fluid of RA patients [73,74,75]. It is conceivable that the increase in ADA is a pathogenic factor, as the increased adenosine deamination will result in reduced bioavailability and decreased AR-mediated inflammation suppression [76]. Furthermore, ADA activity can serve as a useful marker for monitoring the effects of methotrexate as this drug acts in the metabolism of adenosine [77].

The most common form of arthritis is osteoarthritis (OA), which is the most important cause of disability in the elderly. Adenosine signaling plays a critical role in maintaining joint cartilage and can serve as a new therapy for OA. Mice lacking A_2A_AR or CD73 develop spontaneous OA by 16 weeks of age [78]. Exogenous adenosine treatment by intra-articular injection of liposomal suspensions containing adenosine prevents the development of OA in rats. As an approach to extend its half-life, adenosine conjugated to biodegradable nanoparticles prevented the development of OA in a rat post-traumatic model [79]. Recently, in the human TC28a2 cell line, A_2A_AR stimulation increased activation and nuclear localization of FoxO1 and FoxO3, promoted an increase in autophagic flux, improved metabolic function, and reduced apoptosis [80]. All these findings offer evidence that A_2A_ARs may be a useful pharmacological target for OA by promoting chondrocyte and cartilage homeostasis. 

The NF-κB signaling pathway is an important factor involved in the pathogenesis of OA. It has been reported that NF-κB is deregulated by the presence of the A_3_AR agonist CF101 resulting in the reduction of TNF-α in a rat model of OA induced by monosodium iodoacetate. Furthermore, CF101 induces apoptosis of inflammatory cells and acts as a protective agent of cartilage, suggesting that it may be a suitable candidate for the treatment of OA [81]. The safety and efficacy of IB-MECA were also evaluated in a phase II clinical study with patients with knee OA [82]. 

In vitro studies on joint cells have suggested that pulsed electromagnetic field (PEMF) exposure mediates significant protection against the catabolic effect of proinflammatory cytokines and anabolic action that increases matrix synthesis and cell proliferation [83]. Mechanistically, PEMFs were able to mediate the up-regulation of A_2A_AR and A_3_AR in bovine chondrocytes and synoviocytes [84,85]. Thereafter, in human chondrocytes and osteoblasts, stimulation with PEMFs has been observed to potentiate the AR-mediated reduction of prostaglandin E2 (PGE2), IL-6, and IL-8, suggesting their potential in the treatment of inflammatory disorders of the bone and joints [86]. Therefore, PEMFs could be an innovative physiological alternative to the use of AR agonists as they can mediate the effects of tissue-specific agonists without any desensitization and down-regulation [50].

## 4. Chronic Lung Diseases and Pulmonary Inflammation

Chronic lung diseases such as asthma and chronic obstructive pulmonary disease (COPD) are characterized by persistent inflammation and tissue remodeling processes leading to a progressive loss of pulmonary functions [87]. Although the pathogenesis of chronic lung diseases is multifactorial, a common feature among these pathologies is excessive recruitment and aberrant activation of effector cells. These include mast cells, eosinophils, macrophages, neutrophils, lymphocytes, and dendritic cells as well as airway epithelial cells and fibroblasts. Dysregulation of these cells leads to the release of a plethora of mediators that contribute to pulmonary inflammation and remodeling [88,89]. ARs are expressed in all these cell types and various studies indicate that their activation by increased levels of adenosine participates in the pathogenesis of chronic lung diseases [90]. Indeed, elevated adenosine levels were initially reported in bronchoalveolar lavage fluid (BAL) from asthmatics [91,92] and then confirmed in COPD patients [93,94]. As for other chronic lung diseases, there is little evidence of elevated adenosine levels in patients with interstitial lung disease or idiopathic pulmonary fibrosis, although increased concentrations have been found in animal models of these diseases [95,96]. These chronically elevated adenosine levels seem to induce the release of inflammatory mediators leading to tissue injury and fibrosis [90]. The first evidence derived from animal models of airway disease suggested a clinical benefit to the use of A_1_, A_2B_, or A_3_AR antagonists in the treatment of asthma and COPD [97,98]. Subsequent studies have identified the A_2B_AR as the most promising candidate, among AR subtypes, as a target for the treatment of chronic lung diseases. The activation of A_2B_ARs on various pulmonary and immune cell types has in fact been correlated with aberrant cell differentiation and elevated levels of proinflammatory and pro-fibrotic mediators including IL-4, IL-6, IL-8, fibronectin, and TGF-β [99,100,101]. In mice exposed to bleomycin, the most common animal model of pulmonary fibrosis, direct A_2B_AR activation on vascular cells promoted IL-6 and endothelin-1 release. In the same study, genetic removal of the A_2B_AR or treatment with the A_2B_AR antagonist GS-6201 attenuated vascular remodeling and hypertension [100]. In a subsequent study using the bleomycin model, the conditional knockout mice lacking A_2B_AR on myeloid cells presented with attenuated fibrosis, improved lung function, and showed no evidence of pulmonary hypertension compared with control mice exposed to bleomycin. Furthermore, the authors found a reduction of IL-6 and hyaluronan release and a reduced expression of CD206 and arginase-1, markers of alternatively activated macrophages [102]. The implication of adenosine in chronic lung diseases is corroborated by the fact that ADA-deficient mice develop pulmonary inflammation and injury reminiscent of that seen in asthma [103]. ADA-deficient mice exhibited extensive lung mast cell degranulation [103], increased number of alveolar macrophages, and elevated monocyte chemoattractant protein-3 in the bronchial epithelium [104]. Different studies were performed with the aim to elucidate the role of the specific AR subtypes in ADA-deficient mice-induced pulmonary inflammation. When ADA-deficient mice were treated with the antagonist CVT-6883, they exhibited less pulmonary inflammation, fibrosis, and alveolar airspace enlargement with a significant reduction of proinflammatory cytokines and chemokines as well as mediators of fibrosis and airway destruction [105]. The A_3_AR was found to be expressed in eosinophils and mucus-producing cells in the airways of ADA-deficient mice. Genetic deletion A_3_AR or their blockade with the selective antagonist MRS 1523 prevented airway eosinophilia and mucus production [106]. On the contrary, genetic removal of the A_1_AR from ADA-deficient mice resulted in increased pulmonary inflammation, mucus metaplasia, and alveolar destruction along with exaggerated expression of IL-4, IL-13, chemokines, and matrix metalloproteinases [107]. These findings suggested that the A_1_AR plays an anti-inflammatory and/or protective role in the pulmonary phenotype seen in ADA-deficient mice. Similar results were obtained in ADA/A_2A_AR double knockout mice where the authors observed enhanced inflammation comprised largely of macrophages and neutrophils, mucin production in the bronchial airways, and elevated levels of chemoattractant protein-1 and chemokine (C-X-C motif) ligand (CXCL1) in comparison with ADA-deficient mice with the A_2A_AR. This suggested a protective role of A_2A_ARs in pulmonary inflammation [108]. 

Alteration of ARs has been found in chronic lung diseases strengthening the hypothesis of a potential role played by adenosine and its receptors in the pathogenesis of these diseases. In lung parenchyma obtained from subjects with COPD compared with control smokers, binding experiments revealed an increased density of A_1_, A_2A_, and A_3_ARs, while a decrease of A_2B_AR expression. Furthermore, a significant correlation was found between the affinity and density of the ARs and the FEV1/FVC ratio [109]. In a subsequent study, the authors found a significant decrease of A_2B_AR density in BAL macrophages from patients with COPD when compared with healthy smokers [110]. 

As opposed to its detrimental role in the chronic setting, elevated levels of extracellular adenosine promote tissue-protective responses in acute pulmonary inflammation. Most of the studies conducted have identified the A_2A_ and A_2B_ARs as the major factors responsible for the protective action of adenosine in acute pulmonary inflammation [111,112,113,114]. In a model of acute lung injury, aerosolized BAY 60-6583 treatment, an A_2B_AR agonist, was associated with attenuated pulmonary edema, improved histologic lung injury, and dampened lung inflammation. In this model, tissue-specific deletion of A_2B_ARs suggested that alveolar epithelial A_2B_AR signaling contributes to lung protection during acute pulmonary inflammation [115]. In a murine model of lipopolysaccharide (LPS)-induced pulmonary inflammation, A_2B_AR on hematopoietic cells was found to be crucial for the anti-inflammatory effect obtained with the inhibition of SDF-1 receptors CXCR4 and CXCR7 [116]. In another study based on LPS-induced acute lung injury, treatment with adenosine or NECA, an AR non-selective agonist, recovered lung vascular barrier and reduced inflammation [117]. The activation of heme oxygenase 1 by hemin significantly decreased leukocyte migration and chemokine levels into the lung after LPS inhalation, an effect abolished in A_2A_ and A_2B_AR knockout mice [118]. In a mouse model of carrageenan-induced pleurisy, administration of the A_2A_AR agonist CGS 21680 caused a significant reduction of neutrophil infiltration, nitric oxide and cytokine production, NF-κB expression, and PARP activation [119]. 

Apart from A_2A_ and A_2B_ARs, few studies have investigated the role of the other AR subtypes. In a murine model of LPS-induced lung injury, treatment with the A_1_AR agonist 2’-Me-CCPA attenuated polymorphonuclear cells accumulation in the interstitium and alveolar space, microvascular permeability, and reduced TNF-α, IL-6, and CXCL2/3 levels in the BAL [120]. Activation of A_3_AR by the selective agonist Cl-IB-MECA dampened lung dysfunction, inflammation, and neutrophil infiltration after ischemia-reperfusion in wild-type but not A_3_AR knockout mice [121]. In mice exposed to bleomycin, genetic deletion of A_3_ARs resulted in enhanced inflammatory responses associated with an increase of eosinophils [122].

It is worth mentioning that recently, a COVID-19 patient with unresponsive respiratory failure was treated with adenosine for compassionate use, showing a rapid improvement of clinical conditions [123].

## 5. Intestinal Inflammation

Inflammatory bowel diseases (IBDs), including Crohn’s disease and ulcerative colitis, are chronic, progressive inflammatory conditions presenting an overactive intestinal immune system [124]. IBDs are characterized by an overproduction of proinflammatory mediators associated with morpho-functional alterations of the enteric nervous system, and intestinal dysfunctions [125]. Over the years, several studies have emphasized the crucial role of adenosine signaling in the pathophysiology of IBDs [126]. Consistent with the recognized anti-inflammatory role of A_2A_ARs, most studies have identified A_2A_AR stimulation as the most promising approach for the potential treatment of IBDs compared to the pharmacological modulation of the other AR subtypes. In the acute model of rabbit immune colitis, treatment with the A_2A_AR agonist ATL-146e reduced inflammation in the intestinal mucosa by inhibiting proinflammatory cytokines such as TNF-α, INF-γ, and IL-4, and decreasing leukocyte infiltration into the colonic mucosa [127]. In a rat model of experimental colitis, Antonioli and colleagues further elucidated the role of A_2A_AR on colonic motility. In this model, the A_2A_AR antagonist ZM 241385 increased transmural electrical stimulation-induced contractions with a more noticeable effect inflamed respect to control animals, while the A_2A_AR agonist CGS 21680 induced a concentration-dependent reduction of contractile responses [128]. Furthermore, overexpression of A_2A_AR was found in colonic tissues isolated from inflamed animals. The results of a subsequent study by the same group indicated that, while in normal colon, both A_1_ and A_2A_ARs contribute to the inhibitory control of motor functions at the neuronal level, under bowel inflammation, only A_2A_ARs preserved an inhibitory control of colonic neuromotility [129]. More recently, in a rat model of oxazolone-induced colitis, the A_2A_AR agonist PSB-0777 improved body weight, and reduced inflammatory parameters and colonic myeloperoxidase levels [130]. 

Using adoptive T cell transfer studies, Naganuma et al. suggested that A_2A_ARs play a critical role in the T cell-mediated regulation of colitis by suppressing the expression of proinflammatory cytokines while sparing anti-inflammatory activity mediated by IL-10 and TGF-β [131]. In the 2,4,6-trinitrobenzene sulphonic acid (TNBS)-induced chronic model of experimental colitis, the involvement of A_2A_ARs was investigated with the antagonist SCH-442416, suggesting a protective effect of their activation [132]. In mice with toxin A-induced enteritis ATL 313, an A_2A_AR agonist, reduced tissue injury and inflammation by reducing toxin A-induced secretion and edema, mucosal disruption, and neutrophil infiltration [133]. 

More recently, the stimulation of A_2A_ARs with polydeoxyribonucleotide ameliorated the clinical symptoms and weight loss associated with two rat models of experimental colitis represented by intra-colonic instillation of dinitrobenzenesulfonic acid (DNBS) and dextran sulfate sodium (DSS) in drinking water [134]. Polydeoxyribonucleotide promoted the histological repair of damaged tissues, reduced the production of inflammatory cytokines, and decreased myeloperoxidase activity and malondialdehyde.

It was also found that the anti-inflammatory effect of STW 5 (Iberogast^®^), an established herbal combination showing clinical efficacy in functional dyspepsia and irritable bowel syndrome, was dependent upon A_2A_AR activation in a rat-intestinal inflammatory model induced by intraluminal instillation of TNBS [135].

The role of A_2B_ARs in intestinal inflammation is more controversial than that of A_2A_ARs. The proinflammatory role of A_2B_AR activation was suggested by the administration of A_2B_AR antagonist ATL-801 in DSS-treated mice. Compared to mice treated with DSS alone, animals receiving ATL-801 showed a significantly lower extent and severity of colitis with a reduction of IL-6 levels, histological scores, and proliferative indices [136]. The detrimental role of this receptor subtype was further corroborated by the fact that A_2B_AR genetic deletion attenuated colonic inflammation induced by DSS or TNBS [137]. Moreover, a recent study demonstrated that the inhibition of A_2B_AR with the antagonist PSB1115 attenuated intestinal injury in a neonatal rat model of necrotizing enterocolitis [138]. By contrast, an opposite effect was reported in the DSS colitis model, where both the administration of PSB1115 and the genetic deletion of A_2B_AR resulted in an increase in the severity of colitis [139]. 

Various preclinical studies have indicated the activation of A_3_ARs as a promising pharmacological approach for attenuating bowel inflammation [125,140]. In particular, using various experimental models of colitis, A_3_AR agonists were shown to attenuate inflammatory cell infiltration and to reduce proinflammatory mediator levels resulting in an improvement of the intestinal injury [141,142]. In murine DSS colitis, the activation of A_3_ARs attenuated NF-κB activation leading to the reduced expression of TNF-α and IL-1β in colonic epithelia [143]. A more recent study highlighted a down-regulated A_3_AR expression in the colonic mucosa of patients with ulcerative colitis. In cultured colonic mucosal tissue from these patients, the stimulation of A_3_AR with the agonist 2-Cl-IB-MECA significantly decreased TNF-α and IL-1β production and attenuated the NF-κB p65 activation [144]. 

As shown in other inflammatory conditions, the increase of endogenous adenosine levels obtained by blocking catabolic enzymes like ADA or adenosine kinase, or inhibiting nucleoside transporters, also resulted in an improvement in experimental settings of bowel inflammation [77,145,146]. Antonioli et al. showed that inhibiting ADA with EHNA in different mouse models of intestinal inflammation produced beneficial effects characterized by a reduction of proinflammatory cytokines, inflammatory cell infiltration, and a general reduction of colonic damage [147,148,149].

## 6. Neuroinflammation

The central nervous system (CNS), being separated from the periphery by the blood-brain barrier, is characterized by unique features of immune cell distribution and inflammatory responses. Neuroinflammation is mediated by several proinflammatory mediators produced by activated CNS-resident cells such as microglia and astrocytes, which are innate immune cells without direct counterparts in the periphery [150]. In addition, endothelial cells, perivascular macrophages, and other CNS-infiltrating immune cells are also important in interpreting and propagating these inflammatory signals within the CNS [151]. The duration and intensity of the inflammatory signals often determine whether this response is harmful or beneficial for the CNS. Low and transient neuroinflammatory responses can in fact represent important signals for the maintenance of cerebral homeostasis, for the processes of memory and learning [152], and tissue repair and remodeling [153]. By contrast, chronic, uncontrolled inflammation in the CNS has been recognized as a pathological factor for neurodegenerative diseases [154,155,156] as well as for neuropsychiatric disorders [157,158]. Pathological neuroinflammation is associated with activation of glial cells with proinflammatory cytokine and chemokine production, increased blood-brain barrier permeability, infiltration of peripheral immune cells, neuronal damage and death, and neuronal atrophy over time [151]. 

In the CNS, adenosine acts as a neuromodulator. Following neuronal stress and damage, extracellular brain adenosine concentration is dramatically increased resulting in the promotion or attenuation of neuroinflammation. Many of the immunomodulatory effects of adenosine in the CNS are mediated by A_1_ and A_2A_AR subtypes [159,160], although recent evidence also points to the involvement of A_2B_ and A_3_ARs [161,162]. The expression of all four ARs has been detected in astrocytes, oligodendrocytes, and microglia [163]. During neuroinflammation, A_2A_AR stimulates activated microglia to assume their characteristic amoeboid morphology [164]. A composite and regional-specific effect of A_2A_AR antagonist SCH-58261 pretreatment on glial cell activation was observed in a rat model of striatal excitotoxicity obtained by unilateral intrastriatal injection of quinolinic acid [165]. In microglia, A_2A_AR blockage has been suggested to directly potentiated the neuroprotective cannabinoid CB_2_ receptor signaling, likely due to conformational changes within the A_2A_AR-CB_2_ receptor heteromer [166]. In two neonatal rat models of neuroinflammation and microglial activation, the gestational low protein diet model, and postnatal ibotenate intracerebral injections, A_2A_ARs and CD73 were increased and the A_2A_AR antagonist SCH-58261 reduced M1 markers [167]. SCH-58261 also prevented the LPS-induced recruitment of activated microglial cells and the increase in inflammatory cytokines in the hippocampus [168]. In contrast, the stimulation of A_1_ARs inhibited the ATP-induced activation of microglia, likely suppressing the Ca^2+^ influx induced by ATP treatment [169]. In a recent study performed on N13 microglial cells, selective A_1_AR agonists or A_2A_AR antagonists prevented the inflammatory effect induced by a cytokine cocktail. Interestingly, the combined effect of A_1_AR agonists and A_2A_AR antagonists showed a synergistic effect [170].

A_1_AR knockout mice exhibited increased microglial response in an experimental brain traumatic injury model in mice. The attenuated responses induced by A_1_AR stimulation were confirmed in vitro using BV-2 microglial cells [171]. A reduction of microglia activation was also observed stimulating A_3_ARs with the agonist IB-MECA, an effect likely underlining their antinociceptive action [172]. The selective A_3_AR agonist 2-Cl-IB-MECA was effective in controlling microglia reactivity induced by elevated hydrostatic pressure, with potential positive repercussions in glaucoma [173]. In a model of subarachnoid hemorrhage, 2-CI-IB-MECA markedly directed microglia towards the M2, or more precisely M(IL-4) phenotype, reduced inflammation, and improved neurological dysfunction [174]. Marked attenuation of neuroinflammation was also observed with the A_3_AR agonist MRS5980 in a mouse model of traumatic brain injury [162]. Among ARs, the A_2B_AR subtype seems the least involved in the process of neuroinflammation. Nevertheless, A_2B_AR activation was shown to increase IL-6 production and cell proliferation in murine primary microglial cells, an effect involving PLC, PKC-ε, PKC-δ, and p38 pathways, thus suggesting their contribution to microglial activation and neuroinflammation [175]. 

The modulation of neuroinflammation by ARs makes them an attractive pharmacological target for neurodegenerative diseases that share chronic brain inflammation as a ubiquitous common feature, including Alzheimer’s disease [176,177], Parkinson’s disease [178,179], multiple sclerosis [180,181], and Huntington’s disease [182]. Remarkably, an alteration of A_2A_ARs was found in the brain and peripheral blood cells of patients affected by such neurodegenerative diseases [183,184,185,186,187,188].

Experimental autoimmune encephalomyelitis (EAE) is the most commonly used experimental model for the inflammatory demyelinating disease, multiple sclerosis [189]. Compared with wild-type mice, A_1_AR knockout mice developed a more severe form of EAE characterized by worsened demyelination, axonal injury, and enhanced microglial activation [190]. More recently, a similar exacerbation of EAE was found in mice lacking A_2A_ARs [191]. A subsequent work using A_2A_AR knockout mice suggested a dual role for A_2A_ARs in EAE: while providing protection at early stages of the disease by exerting anti-inflammatory effects on T cells, A_2A_ARs seem to be detrimental during later stages contributing to tissue damage within the inflamed CNS [192]. Using a different approach, mice lacking CD73 were resistant to EAE. According to the authors, the protection was not caused by a deficiency in T cell responsiveness and CD73 must be expressed either on T cells or in the CNS for disease induction [193]. The treatment with the A_2A_AR antagonist SCH-58261 protected wild-type mice from EAE by inhibiting the entry of lymphocytes into the CNS. In a subsequent work by the same authors, results obtained with bone marrow chimeric mice revealed that A_2A_AR expression on nonimmune cells is required for efficient EAE development, while A_2A_AR expressed on lymphocytes is essential for limiting the severity of the inflammatory response [194]. The confounding role of A_2A_ARs in EAE seems to be related to the identification of the best therapeutic window: depending on the treatment period, both A_2A_AR agonist and antagonist have been proved to protect against EAE development [195,196,197].

## 7. Immunity, Inflammation, and Cancer

Cancer is a complex disease, caused by multiple cellular dysregulations [198]. The immune system is involved in all stages of cancer progression. In physiological conditions, the immune system is able to remove abnormal cells, but when cancer cells escape the immune surveillance and start growing, immune cells infiltrate the tumor [199]. The cancer immune-escaping mechanism uses different factors. The involvement of adenosine in controlling inflammation and preventing exaggerated immune response is well established. Some kinds of tumors are infiltrated by different immune cells and can use adenosine as an immunosuppressive agent to block immune response versus cancer cells [198]. Indeed, in cancer cells there is commonly an augmented level of adenosine accompanied by overexpression of CD39 and CD73 thus limiting the immune cell activity in the tumor microenvironment [200,201,202,203,204]. Many studies demonstrated that the intratumoral overexpression of ectonucleotidases could be principally due to hypoxia and inflammation [205,206]. The activation of HIF-1α, as well as the signaling pathways triggered by proinflammatory mediators (IL-1β, IL-6, TNF-α, TGF-β), may boost CD39 and CD73 levels [207,208,209,210,211,212]. It is now understood that T cells are a key player in tumor control; the elevated levels of adenosine in the tumor can potently impair T cells mediated antitumor response by inducing accumulation of intracellular cAMP [199,213]. 

In the tumor microenvironment, there are two types of infiltrating macrophages, called tumor-associated macrophages: classically activated macrophages (M1) and alternatively activated macrophages (M2) [214]. Studies so far conducted reported that M1 predominately expresses A_2A_ARs, while A_2B_ARs are responsible for the alternative macrophages’ activation into the M2 phenotype [215]. Adenosine exerts many suppressive effects on antitumoral M1 macrophages mostly through A_2A_AR activation, which decreases proinflammatory cytokine release while increasing the secretion of IL-10, an anti-inflammatory mediator [198]. M2 macrophages exhibit protumoral functions; in particular, a subtype of M2, called M2d, is activated by A_2A_ARs [216]. As a result, they release proangiogenic factors such as VEGF and IL-10. In addition, IL-10 endorses the polarization of Th17 and Treg leading to inflammatory response inhibition. Evidence collected so far suggests that M2 macrophages affect tissue repair and matrix remodeling hence promoting tumor growth, angiogenesis, and metastasis development [217,218].

Similar to macrophages, neutrophils are a crucial player in the interaction between the immune system and cancer cells. When infiltrated in the tumor, they fine-tune adaptive cells enrollment through cytokine and chemokine production [219]. Neutrophils can be subjected to two different polarizations, similarly to macrophages, achieving an antitumor phenotype (N1) or a protumor (N2) phenotype. The major difference between these two cell types is their capacity for the production of pro- or anti-inflammatory cytokines and that N2 cells are proangiogenic and prometastatic [220]. Several studies on tumor infiltrated neutrophils reported that adenosine hinders the migration of these cells from the bloodstream to the inflammatory site, blocking their adhesion to endothelial cells. Moreover, the activation of A_2A_ARs on neutrophils inhibits proinflammatory cytokine and chemokine production such as TNF-α, CCL3, CCL4, and others, causing an inefficient enrollment of immune cells [221]. Other studies showed that, besides A_2A_Ars, A_3_AR activation is also involved in the inhibition of neutrophil degranulation thus reducing their proinflammatory potential [222].

The adenosine in the tumor microenvironment also acts on natural killer cells. A_2A_ARs stimulation interrupts the maturation process of these cells, which can result in natural killer cell death [198]. Indeed, in A_2A_AR knockout mice a greater amount of mature natural killer cells has been found associated with a reduced number of immature cells. Furthermore, the mature natural killer cells showed an increased ability to control tumor initiation and growth [223]. A recent paper reports that infiltrated natural killer cells are modulated by cancer cells through CD137 engagement on the cell surface, inducing the translocation of vesicles containing CD73 on the natural killer cell membrane, thus promoting the secretion of IL-10 and TGF-β [224]. IL-10 inhibits the proliferation of CD4+ T cells and its IFN-γ production, thus blocking adaptive antitumor response, and CD73 on natural killer cells increases adenosine concentration in the tumor microenvironment leading to immunosuppression [224]. 

In recent studies, the role of mast cells in the tumor microenvironment has been investigated. Apart from their role in allergic reactions and inflammation, they are also involved in wound healing and angiogenesis; thus, they can result in protumoral cells. Indeed, mast cells can be triggered by direct contact with cancer cells when they infiltrate the tumor [225,226]. This activation results in IL-8, IL-6, and VEGF secretion [221]. These mediators promote new blood vessels angiogenesis supporting tumor growth and metastasis. Both mechanisms involve CD73 activation on mast cells and autocrine signaling of adenosine through A_3_ARs stimulation [225,226].

Adenosine affects the normal function of dendritic cells. They are antigen-presenting cells responsible for the activation of T cells and induction of their differentiation [198]. Dendritic cells derive from bone marrow monocyte-dendritic cell progenitor, which differentiate into common the dendritic cell’s precursor. During inflammatory reactions caused by cancer or infections, it was observed, both in vivo and in vitro, that monocytes can give rise to a subset of dendritic cells called monocyte-derived dendritic cells [227]. Mature dendritic cells express A_2A_ARs, thus their stimulation with adenosine or agonists resulted in increased levels of intracellular cAMP and inhibited the production of IL-12. In addition, dendritic cells maturated in the presence of adenosine produce IL-10 and have a reduced capacity of induction of Th1 [227]. Studies performed on differentiation of human monocytes, mouse peritoneal macrophages, and hematopoietic progenitor cells into myeloid dendritic cells, in the presence of increased levels of adenosine, revealed an impaired activation and function of resulting dendritic cells, as they fail to activate naive T cells and they produce anti-inflammatory and angiogenic factors [23]. This adenosine-mediated effect was due to the activation of A_2B_ARs [23]. 

Given the harmful role of A_2A_ and A_2B_AR stimulation on tumor-infiltrating immune cells which suppress their antitumor effect, many studies have been conducted using antagonists, in order to restore antitumor immunity and enhance the efficacy of cancer immunotherapies [228]. Interestingly, A_2A_AR antagonists ZM241385 and SCH58261 were able to restrain primary tumor growth, prevent negative T cells signaling, and inhibit angiogenesis in a mice model of non-small cell lung cancer [229]. Three A_2A_AR antagonists are currently undergoing phase I/II clinical trials as single agents for the treatment of solid tumors: CPI-444 (NCT02655822), AZD4635 (NCT02740985), and NIR178 (NCT02403193, NCT03207867), respectively. A_2B_AR signaling in tumor cells themselves further promotes their survival and metastasis [228]. Notably, administration of A_2B_AR antagonists decreases tumor growth in mice tumor model and metastasis [230,231]. In vivo, the block of A_2B_ARs increases CD8^+^ T cells, natural killer cells, and the production of TNF-α and IFN-γ in the tumor microenvironment, accompanied by reduced levels of IL-10, VEGF, and angiogenesis [232,233,234]. From these encouraging preclinical results, a dose-escalation phase I clinical trial (NCT03274479) administering PBF-1129, a selective A_2B_AR antagonist, in patients with advanced non-small cell lung cancer has been started [212]. 

## 8. Conclusions

Adenosine, through interaction with its four receptor subtypes, is a ubiquitous and powerful modulator of inflammatory processes affecting almost all physiological and pathophysiological functions. All immune cells express ARs and respond in a different manner to adenosine, the concentration of which varies enormously in stressful situations including inflammation. Considering that inflammation is a hallmark of many chronic diseases, both in the periphery and in the CNS, the modulation of ARs has a huge potential as a therapeutic strategy. However, the wide distribution of ARs and the multiple functions of adenosine in the body was supposed to be a limiting factor for the drug development acting on this system. Indeed, once in the clinic, AR ligands have often shown problems of low efficacy and/or manifestation of adverse effects. Nevertheless, the recent approval of the A_2A_ARs antagonist istradefylline for Parkinson’s disease and the encouraging results obtained so far on cancer immunotherapy has again turned the spotlight on the adenosine system as a drug target. Great efforts are being made to identify new and more selective AR ligands as well as adenosine-regulating agents capable of modulating the concentration of endogenous adenosine. As a matter of fact, numerous clinical trials are currently being performed on AR ligands (Table 1).

In conclusion, the adenosinergic system has considerable therapeutic potential in all pathological states and in particular in those diseases where regulation of inflammation and modulation of the immune system is required.

## Figures and Tables

**Table 1:** Current clinical trials of AR ligands (data from ClinicalTrials.gov, accessed 15 July 2021).

Compound	Pharmacological Behavior	Condition	Phase	Identifier
Regadenoson	A_2A_AR agonist	Lung Transplantation	1	NCT03072589
Regadenoson	A_2A_AR agonist	Lung Transplantation	1	NCT04521569
Regadenoson	A_2A_AR agonist	Gliomas	1	NCT03971734
Regadenoson	A_2A_AR agonist	COVID-19	1/2	NCT04606069
Regadenoson	A_2A_AR agonist	Heart Transplant	4	NCT03102125
Ciforadenant	A_2A_AR antagonist	Incurable Cancers	1	NCT02655822
Ciforadenant	A_2A_AR antagonist	Multiple Myeloma	1	NCT04280328
Ciforadenant	A_2A_AR antagonist	Advanced Cancers	1	NCT03454451
Etrumadenant (AB928)	A_2A_ and A_2B_ AR antagonist	Metastatic Castrate Resistant Prostate Cancer	1b/2	NCT04381832
Etrumadenant (AB928)	A_2A_ and A_2B_ AR antagonist	Head and Neck Cancers	1	NCT04892875
Etrumadenant (AB928)	A_2A_ and A_2B_ AR antagonist	Non-Small Cell Lung Cancer	2	NCT04791839
Etrumadenant (AB928)	A_2A_ and A_2B_ AR antagonist	Non-Small Cell Lung Cancer	2	NCT04262856
Etrumadenant (AB928)	A_2A_ and A_2B_ AR antagonist	Advanced Cancers	1	NCT03629756
Etrumadenant (AB928)	A_2A_ and A_2B_ AR antagonist	Lung Cancer	1	NCT03846310
Etrumadenant (AB928)	A_2A_ and A_2B_ AR antagonist	Triple-Negative Breast Cancer or Gynecologic Malignancies	1	NCT03719326
Etrumadenant (AB928)	A_2A_ and A_2B_ AR antagonist	Colorectal Cancer	1/2	NCT04660812
Etrumadenant (AB928)	A_2A_ and A_2B_ AR antagonist	Prostate Cancer	2	NCT03821246
Etrumadenant (AB928)	A_2A_ and A_2B_ AR antagonist	Metastatic Pancreatic Ductal Adenocarcinoma	1/2	NCT03193190
Etrumadenant (AB928)	A_2A_ and A_2B_ AR antagonist	Metastatic Colorectal Cancer	1/2	NCT03555149
PBF-1129	A_2B_AR antagonist	Advanced Non-Small Cell Lung Cancer	1	NCT03274479
Namodenoson (CF102)	A_3_AR agonist	Non-Alcoholic Steatohepatitis	2	NCT04697810
Piclidenoson (IB-MECA)	A_3_AR agonist	COVID-19	2	NCT04333472
CF101 (IB-MECA)	A_3_AR agonist	Plaque Psoriasis	3	NCT03168256

## Data Availability

Not applicable.

## Abbreviations

AC	Adenylate cyclase
ADA	Adenosine deaminase
ADP	Adenosine diphosphate
AMP	Adenosine monophosphate
ARs	Adenosine receptors
ATP	Adenosine triphosphate
BAL	Bronchoalveolar lavage fluid
cAMP	Cyclic adenosine monophosphate
CD39	Ectonucleoside triphosphate diphosphohydrolase
CD73	Ecto-5′-nucleotidase
CNS	Central nervous system
COPD	Chronic obstructive pulmonary disease
COX-2	Cyclooxygenase 2
CXCL	Chemokine (C-X-C motif) ligand
DNBS	Dinitrobenzenesulfonic acid
DSS	Dextran sulfate sodium
EAE	Experimental autoimmune encephalomyelitis
ENT	Equilibrative nucleoside transporter
GC	Germinal center
GPCR	G protein-coupled receptor
IBDs	Inflammatory bowel diseases
IDO-1	Indoleamine 2,3 dioxygenase
IFN-γ	Interferon γ
IL	Interleukin
LPS	Lipopolysaccharide
MIP	Macrophage inflammatory protein
NF-kB	Nuclear factor κ-light-chain-enhancer of activated B cells
OA	Osteoarthritis
PEMFs	Pulsed electromagnetic fields
PGE2	Prostaglandin E2
RA	Rheumatoid arthritis
ROS	Reactive oxygen species
TGF-β	Tissue growth factor β
TNBS	2,4,6-trinitrobenzene sulphonic acid
TNF-α	Tumor necrosis factor α
VEGF	Vascular endothelial growth factor
